# Supplementary material for: Complexome profiling of the Chlamydomonas psb28 mutant reveals TEF5 as an early PSII assembly factor
Source: Plant Cell. 2025 Mar 17;37(6):koaf055. doi: 10.1093/plcell/koaf055 (PMC12164590; doi:10.1093/plcell/koaf055)
Supplement: koaf055_Supplementary_Data [file koaf055_supplementary_data.zip › psb28_interactive.zip!/plots/intensity/qualityPlots/peptides/_Cre06.g278195.t1.1.html]

### Cre06.g278195.t1.1

Trivial name:   
SeqCoverage [median]: 45.40  
Mass: 14.08 kDa  
  
 

| Sequence | MissedCleavage | Mass [Da] | wtIntensity | muIntensity | Score |
| --- | --- | --- | --- | --- | --- |
| ALLGDDQR | 0 | 886.45 | 17073.21 | 32845.92 | 165.11 |
| ALLGDDQRLK | 1 | 1127.63 | 892.50 | 1685.23 | 64.04 |
| EGDGMKIESTP | 1 | 1162.52 | 357.65 | 1273.40 | 96.95 |
| FLEDEQPKDPEQTK | 1 | 1702.81 | 434.54 | 3226.84 | 110.92 |
| FLEDEQPKDPEQTKQDLIEK | 2 | 2429.20 | 0.00 | 23412.38 | 112.74 |
| IAQLNASIDEVAAQLK | 0 | 1682.92 | 4248.69 | 6932.21 | 220.53 |
| QDLIEK | 0 | 744.40 | 0.00 | 1049.67 | 97.07 |
| VKEGDGMKIESTP | 2 | 1389.68 | 2286.61 | 4371.06 | 71.51 |
|  |  |  | 25293.2 | 74796.7 |  |
